# Synthesis and Biological Evaluation of New CRH Analogues

**DOI:** 10.1155/2010/252348

**Published:** 2010-06-28

**Authors:** Spyridon Papazacharias, Vassiliki Magafa, Nicole Bernad, George Pairas, Georgios A. Spyroulias, Jean Martinez, Paul Cordopatis

**Affiliations:** ^1^Laboratory of Pharmacognosy and Chemistry of Natural Products, Department of Pharmacy, University of Patras, 26500 Patras, Greece; ^2^Institut des Biomolécules Max Mousseron (IBMM), UMR-CNRS, Faculté de Pharmacie, Universités Montpellier 1 et 2, 15 Av. C. Flahault, 34093 Montpellier, France

## Abstract

A series of 7 new human/rat Corticotropin Releasing Hormone (h/r-CRH) analogues were synthesized. The induced alterations include substitution of Phe at position 12 with D-Phe, Leu at positions 14 and 15 with Aib and Met at positions 21 and 38 with Cys(Et) and Cys(Pr). The analogues were tested regarding their binding affinity to the CRH-1 receptor and their activity which is represented by means of percentage of maximum response in comparison to the native molecule. The results indicated that the introduction of Aib, or Cys derivatives although altering the secondary structure of the molecule, did not hinder receptor recognition and binding.

## 1. Introduction

Ever since its identification by Vale et al. [1], Corticotropin Releasing Hormone (CRH) has proven to be a major neuromodulator responsible not only for the secretion of ACTH by the anterior pituitary gland but also for the regulation of the endocrine, autonomic, immunological and behavioral responses to stress [2–4]. Furthermore, this 41-amino acid neuropeptide displays a plethora of additional roles in either physiological homeostasis or pathogenic manifestations varying from the well-established implication on neuropsychiatric disorders [5, 6] to the yet to be clarified actions on various forms of cardiovascular diseases [7] obesity [8] or gut motility [9], among others. A series of studies demonstrating the extent and perplexity of the roles and implications of CRH led to the identification of other peptides appearing to possess a similar role [10–13].

Both the isolation and characterization of the CRH family receptors has been achieved revealing two receptor subtypes, one of them appearing as three splice variants (CRH-R1, CRH-R2_*α*_, CRH-R2_*β*_, and CRH-R2_*γ*_). A binding protein (CRH-BP) was also described [14–18].The most-studied receptor types are CRH-R1, CRH-R2_*α*_, and CRH-R2_*β*_ not only due to their vast distribution but also for their implication in physiological functions or disorders of great importance. CRH-R1 is broadly distributed in the brain and the pituitary gland but also appears in a number of peripheral tissues [19]. It possesses a critical role in mediating the hypophysiotropic action of CRH [20] but furthermore is involved in anxiogenic behaviours, depression and anxiety [21], anorexia and bulimia, drug seeking and withdrawal, and seizure [22, 23]. CRH-R2_*α*_ is mainly a brain receptor whereas CRH-R2_*β*_ is largely expressed in the periphery. The role of CRH-R2 is more diverse since it extends from “stress-coping” responses (anxiolysis, hypotension) to gastric emptying, regulation of energy expenditure, anti-inflammatory, and other actions of CRH [24]. However, further and complete understanding of the hormone-receptor interaction on its chemical basis is essential mainly since it can provide a solid basis for the development of CRH analogues with potent therapeutical implications. 

Two distinct categories of CRH receptor ligands (of either agonistic or antagonistic character) have been developed, namely, peptide and nonpeptide ligands. The latter are unambiguously a large and diverse family but only few of these small molecules entered clinical development and still none of them found its way to the market [25]. The former category is more likely to yield clear information regarding the interaction between the peptide and its receptor. 

Towards this goal, extensive SAR studies have been carried out. Single amino acid modification studies identified the key regions of the peptide for agonist/antagonist properties as well as for the receptor binding affinity. Based on these studies, the assignment of the molecule's secondary structure was feasible leading to the establishment of an *α*-helical structure (showing high amphiphilicity) for CRH-related peptides as the preferred conformation [9]. Notably, the helical nature of the molecule varies from 20% in solution to 80% upon binding [26].

A hypothesis was that stabilization of the *α*-helix could affect positively the activity of either agonists or antagonists. Subsequent studies affirmed this conjecture establishing that conformationally restrained analogues present increased potency. In addition, modifications on hydrophilicity/hydrophobicity or acidity/basicity of some residues were crucial in altering the potency of an analogue [26].

Introduction of D-amino acids leading to stabilization of *β*-turns, can provide analogues with increased potency but not all residues are susceptible to such an alteration, leading even to the opposite effect. However, the D-Phe^12^ substitution has proven to be a favorable one especially combined with other advantageous modifications such as the replacement of Met at positions 21 and 38 with Nle. Thus, it has been shown that [D-Phe^12^]CRH is twice as potent and [D-Phe^12^, Nle^21, 38^]CRH_21-41_ is 15 times more potent than **α**-helical CRH [26].

The present study presents the synthesis of 7 new h/r-CRH analogues bearing minor modifications on the key amino acids side chain, regarding their electrochemical nature (Table 1). Specifically, we discuss the replacement of Leu at positions 14 and 15 with *α*-Aminoisobutyric acid (Aib), which apart from introducing a bend in the peptide backbone can stabilize the *a*-helix structure [27, 28], and also the replacement of Met at positions 21 and 38. The amino acids used for the latter modification are Cysteine(Ethyl) [Cys(Et)], an isoster of Met and Cysteine(Propyl) [Cys(Pr)] that possesses a slightly more hydrophobic side chain than Cys(Et). The analogues were tested regarding their ability to induce the formation of intracellular cAMP in comparison to natural CRH measured by means of luciferase activity.

## 2. Experimental

### 2.1. Materials

2-chlorotrityl-chloride resin bearing a Rink-Bernatowitz linker and 9-Fluorenylmethoxycarbonyl (Fmoc)-protected amino acids were supplied by CBL Patras. All solvents and reagents used for solid-phase peptide synthesis were purchased from Bachem AG and Novabiochem and were used without further purification being of analytical quality. Nonnatural amino acid derivatives Cysteine (Ethyl) [Cys(Et)] and Cysteine (Propyl) [Cys(Pr)] were prepared according to literature [29].

### 2.2. Peptide Synthesis and Purification

Synthesis of the analogues was performed via Fmoc solid phase methodology [30] utilizing either Rink Amide MBHA resin [31] or 2-chlorotrityl-chloride resin [32] bearing a Rink-Bernatowitz linker [33] to provide the peptide amide. The side-chain protection used for the Fmoc-protected amino acids was the trityl group (Trt) for His, Asn, and Gln, the *tert*-butyl group (Bu*^t^*) for Asp, Tyr, Glu, Ser and Thr, the *tert*-butyloxy-carbonyl group (Boc) for Lys and the 2,2,4,6,7-pentamethyl-dihydrobenzofuran-5-sulphonyl group (Pbf) for Arg. Stepwise synthesis of the peptide analogues was preferred to convergent synthesis due to the lack of Gly or Pro moieties at convenient positions in the amino acid sequence. The amino acids were coupled at three-fold excess using diisopropylcarbodiimide/1-hydroxybenzotriazole (DIC/HOBt) [34, 35] in Dimethylformamide (DMF) and, if necessary, 2-(1H-benzotriazole-1-yil)-1,1,3,3-tetramethyluroniumhexafluorophosphate (TBTU)/HOBt/diisopropyl ethylamine (DIEA) in DMF [36]. After 2 hours coupling time at room temperature, the ninhydrin test [37] was performed to estimate the completeness of the reaction with the exception of coupling on a Pro residue where the Chloranil test was employed to confirm reaction's completion point [38]. Fmoc groups were removed by treatment with 20% piperidine in DMF for 5 minutes followed by a prolonged treatment with the same solution for 20 minutes to ensure complete removal. The final cleavage of the peptide from the solid support together with the removal of the side-chain protecting groups was accomplished by treatment with a solution (15 mL/g peptide resin) of trifluoroacetic acid (TFA)/1,2-ethanedithiol/anisole/triethylsilane/water (94 : 2.5 : 0.5 : 1.5 : 1.5, v/v) for 4 hours at room temperature. The obtained mixture underwent solvent evaporation followed by anhydrous ethyl ether precipitation to yield the final crude peptide.

**Figure 1:**
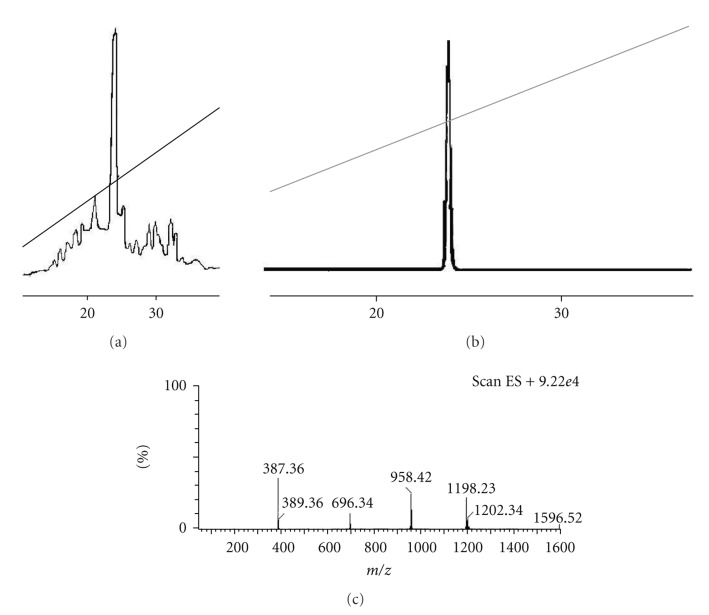
(a) Analytical HPLC chromatogram of crude analogue [D-Phe^12^, Cys(Pr)^21^, Cys(Pr)^38^]CRH previously chromatographed on a semipreparative C18 column. (b) Analytical HPLC chromatogram of analogue [D-Phe^12^, Cys(Pr)^21^, Cys(Pr)^38^]CRH upon rechromatography on the same column. (c) Mass spectra of analogue [D-Phe^12^, Cys(Pr)^21^, Cys(Pr)^38^]CRH resulting from ESI-MS analysis [MW_calc_ = 4785.58; (M+4)_obs_^4+^/4=1198.2; (M+3)_obs_^3+^/3=1596.5].

All the products were purified by gel filtration chromatography on Sephadex G-25 (fine) using 25% acetic acid as eluent. Final purification was achieved by preparative high performance liquid chromatography (HPLC, Pharmacia LKB-2250) on reversed-phase support C-18 with a linear gradient from 30 to 85% acetonitrile (0.1% TFA) for 35 minutes at a flow rate 1.5 mL/min and UV detection at 230 and 254 nm. The appropriate fractions were pooled and lyophilized. Analytical HPLC (Pharmacia LKB-2210) equipped with a Nucleosil 100 C_18_ column (5 *μ*m particle size; 250 × 4.6 mm) produced single peaks with at least 98% of the total peptide peak integrals. The solvent system used was the same as that for the semipreparative HPLC. All products gave single spots on thin layer chromatography (TLC, Merck precoated silica gel plates, type G_60_-F_254_) in the solvent systems: (A) 1-butanol: acetic acid: water (4 : 1: 5, upper phase) and (B) 1-butanol: acetic acid: water: pyridine (15 : 3: 10 : 6). The final characterization of the peptide sequence was achieved by Electrospray Ionisation-Mass Spectrometry (ESI-MS, Micromass-Platform LC instrument). An example of analytical HPLC-chromatograms and ESI-MS spectrum are shown in Figure 1 for analogue **7**. The physiochemical properties of the new analogues are summarized in Table 2.

### 2.3. Biological Assays

The capacity of CRH and related peptide analogues to stimulate cyclic AMP generation was monitored in LLC-PK1 cells cotransfected with cDNA of CRH-R1 and CRE. For transfection the method of electroporation was applied and 40 millions of LLC-PK1 cells were prepared and washed in cytomix 1X transfection medium. The electroporation medium consisted of 1000 *μ*L of cytomix 2X, 864 *μ*L of sterilized water, 80 *μ*L of ATP 50 mM, 3.2 mg of glutathion, 20 *μ*g of CRH-R1 cDNA and 20 *μ*g of CRE cDNA. 500 *μ*L of the above solution were transferred in the electroporation device and after remaining for 10 minutes a standard growth medium was added (DMEM without phenol red with glutamine, antibiotics, and 10% FCS) to a total volume of 50 mL. The suspension was distributed in 8 plates of 24 wells each, by adding 1 mL per well, and was allowed to pre-incubate for 24 hours. The growth medium was aspirated and replaced with fresh one followed by another preincubation for 24 hours. The cells were then challenged with graded concentrations (10^−6^ to 10^−11^ M) of the test peptides and incubated for 7 hours at 37°C. After that, the medium with the added peptides was removed, the cells were washed twice with PBS 1X, 100 *μ*L of lysis buffer 1X per well were added, the plates were left at room temperature for 30 minutes and then stored at −80°C overnight. The luciferace activity was measured with the use of a luminometer after the addition of 50 *μ*L of lysed cell preparation and 50 *μ*L of bioluminescence medium per well in a 96 well plate luminometer.

## 3. Results

### 3.1. Peptide Synthesis and Purification

All analogues shown in Table 2 were synthesized either manually or automatically on the Rink Amide MBHA resin or the 2-chlorotrityl-chloride resin bearing a Rink-Bernatowitz linker as solid support by using standard coupling procedures and Fmoc/Bu*^t^* strategy. The overall yield of the syntheses of the CRH analogues was in the range 33–41% (calculated on the amount of linker initially coupled to the resin). Higher yields were obtained using the Rink Amide MBHA as solid support. 

The synthetic procedure did not present significant complications due to the highly helical nature of the analogues despite the considerable size of the CRH analogues. ESI mass spectrometry confirmed that the purified products were indeed the desired peptides and analytical HPLC revealed a purity of over than 98% for the synthetic analogues.

### 3.2. Biological Activity

The results of the biological evaluation regarding the synthesized analogues are set out in Tables 3 and 4. The IC_50_ values (Table 3) indicate a not very wide but nonetheless notable variety in the affinity to the receptor for the studied analogues. Specifically, three of the analogues, namely, [D-Phe^12^, Aib^15^]CRH, [D-Phe^12^, Cys(Et)^21^, Cys(Pr)^38^]CRH, and [D-Phe^12^, Cys(Pr)^21^, Cys(Et)^38^]CRH, present a higher affinity to the receptor (IC_50_=8.4 nM, IC_50_=7.0 nM and IC_50_=3.4 nM, resp.) which is approximately 2, 2,5, and 5 times that of the natural h/r-CRH (IC_50_=17.3 nM). [D-Phe^12^, Aib^14^]CRH is the analogue that presents the higher IC_50_ value (226 nM) indicating a smaller affinity to the receptor, roughly 13 times lower than that of the hormone. The other three analogues appear to have the same ([D-Phe^12^, Cys(Pr)^21^, Cys(Pr)^38^]CRH, IC_50_=17.3 nM) or slightly reduced ([D-Phe^12^, Cys(Et)^21^]CRH, IC_50_=28.5 nM and [D-Phe^12^, Cys(Pr)^38^]CRH, IC_50_=35.0 nM) affinity compared to CRH.

Regarding the activity of the analogues which is represented by means of percentage of maximum response compared to that of h/r-CRH (Table 4), the only analogue that presents a higher percentage is [D-Phe^12^, Cys(Et)^21^]CRH (full agonist, 110% response max/CRH). All other analogues are partial agonists and induce a maximum response that varies between 60 and 80 per cent of the maximum response induced by CRH.

## 4. Discussion

The design and synthesis of the seven analogues was based on observations and results already mentioned above and likely to yield products with desirable properties. D-Phe^12^ substitution was a modification present in all analogues since it brings only a positive contribution to the potency and the activity of the analogue. Substitution of Leu at positions 14 and 15 with Aib was based on studies demonstrating that *α*-aminoisobutyric acid is a helix-promoter/stabilizer, which increases the helix propensity of a peptide fragment towards, either *α*- or 3_10_- helix, by restriction of the backbone conformational freedom. Aib, on the other hand, apart from its tendency to form constrained helices, is also known for its ability to bend helical peptides. The conformational features of the analogue [D-Phe^12^, Aib^15^]CRH have already been studied through 1D and 2D *J*-correlated ^1^H NMR spectroscopy [39].

Biological evaluation of the two Aib containing analogues revealed that they both present the same activity which is 60 percent that of the natural hormone and thus, they appear to be partial agonists of CRH. However, when the IC_50_ values are taken under consideration, the two analogues significantly vary regarding their affinity to the receptor. Although the only difference in their secondary structure is the position of the Aib moiety (14 or 15) and furthermore, the two positions are adjacent, [D-Phe^12^, Aib^15^]CRH presents a roughly 27 times higher affinity than [D-Phe^12^, Aib^14^]CRH. An NMR study of [D-Phe^12^, Aib^14^]CRH and consequent comparison with the results already extrapolated for [D-Phe^12^, Aib^15^]CRH is the best means to clarify the effect of the Aib substitution on the molecule conformation and consequently on its binding to the receptor. All the same, a primary estimation could be that the positions 14 and 15 although important for binding to the receptor might not belong to the region that is responsible for the hormone's action since no alteration is observed on the biological activity for the two analogues. 

Cys(Et) and Cys(Pr) are two nonnatural amino acid residues that have already been used in the synthesis of peptides (e.g., Angiotensin II) [40] yielding interesting results. The fact that Cys (S-Et) is an isoster of Met along with the enhanced potency of the analogues with substituted Met^21^ or Met^38^ were basically taken into account for the design of the analogues. Cys (S-Pr) was used since it bears a side-chain that on one hand possesses the S atom present in Met and on the other hand has a longer aliphatic unit than Met or Cys(Et). Two of the analogues were monosubstituted regarding the Met residues, [D-Phe^12^, Cys(Et)^21^]CRH, and [D-Phe^12^, Cys(Pr)^38^]CRH, and three had a double substitution of Met, [D-Phe^12^, Cys(Et)^21^, Cys(Pr)^38^]CRH, [D-Phe^12^, Cys(Pr)^21^, Cys(Et)^38^]CRH and [D-Phe^12^, Cys(Pr)^21^, Cys(Pr)^38^]CRH. 

Both [D-Phe^12^, Cys(Et)^21^]CRH and [D-Phe^12^, Cys(Pr)^38^]CRH present reduced affinity to the receptor compared to CRH, which is approximately half that of the hormone. Notably, when these modifications are combined in the [D-Phe^12^, Cys(Et)^21^, Cys(Pr)^38^]CRH analogue, the affinity appears to enhance significantly. However, this combination does not have the same impact on the activity of the molecule. [D-Phe^12^, Cys(Et)^21^]CRH is the only of the studied analogues that presents a higher than CRH activity (110% that of the hormone) whereas [D-Phe^12^, Cys(Pr)^38^]CRH shows reduced activity (60% that of CRH). The activity of [D-Phe^12^, Cys(Et)^21^, Cys(Pr)^38^]CRH, although slightly higher than that of the latter monosubstituted analogue is considerably lower than that of the former one. This could mean that the negative effect of Cys(Pr)^38^ is more prominent than the positive one of Cys(Et)^21^. As for the affinity, no safe assumptions can be made until further NMR studies are performed on these molecules.

The results regarding [D-Phe^12^, Cys(Pr)^21^, Cys(Pr)^38^]CRH indicate that double substitution with Cys(Pr) does not affect the affinity of the molecule to the receptor and decreases only slightly its activity. When the Cys(Pr) residue is applied at position 38 the IC_50_ value is negatively affected and the molecule shows a 40% decrease in activity compared to CRH. The reversal of this image with the application of Cys(Pr)^21^ could be attributed to a positive effect of this moiety on the molecule but this assumption can be confirmed only by the study of the [D-Phe^12^, Cys(Pr)^21^]CRH analogue that remains to be performed.

The analogue presenting the lowest IC_50_ value is [D-Phe^12^, Cys(Pr)^21^, Cys(Et)^38^]CRH, indicating a 5-fold increased affinity to the receptor compared to the native hormone and also to the [D-Phe^12^, Cys(Pr)^21^, Cys(Pr)^38^]CRH. Regarding the two CRH analogues it could be assumed that the difference in affinity is connected with the residue at position 38, where the Cys(Et) substitution appears to be more favorable compared to Cys(Pr) substitution. 

Considering all the aforementioned remarks, the conclusions are (i) regarding the introduction of Aib, position 15 is preferable to position 14 since it yielded analogues with higher affinity to the receptor; (ii) although the introduction of Aib modifies the secondary structure and can alter significantly the affinity to the receptor, the Aib containing analogues can still be characterised as partial agonists; (iii) affinity to the receptor and activity do not necessarily coincide and analogues that excel in the one may fall short to the other; (iv) simple or combined modifications with Cys(Et) or Cys(Pr) in position 21 or/and position 38 did not hinder receptor recognition leading, in the most of the cases, to partial agonists with the exception of the analogue [D-Phe^12^, Cys(Et)^21^]CRH which presents full agonistic activity.

## Figures and Tables

**Table 1:** Structure of synthesized CRH analogues.

	Human/Rat CRH	Analogue 1	Analogue 2	Analogue 3	Analogue 4	Analogue 5	Analogue 6	Analogue 7
1	S							
2	E							
3	E							
4	P							
5	P							
6	I							
7	S							
8	L							
9	D							
10	L							
11	T							
12	E	D-phe	D-phe	D-phe	D-phe	D-phe	D-phe	D-phe
13	H							
14	L	Aib						
15	L		Aib					
16	R							
17	E							
18	V							
19	L							
20	E							
21	M			Cys(Et)		Cys(Et)	Cys(Pr)	Cys(Pr)
22	A							
23	R							
24	A							
25	E							
26	Q							
27	L							
28	A							
29	Q							
30	Q							
31	A							
32	H							
33	S							
34	N							
35	R							
36	K							
37	L							
38	M				Cys(Pr)	Cys(Pr)	Cys(Et)	Cys(Pr)
39	E							
40	I							
41	I							

**Table 2:** Physicochemical properties of h/r-CRH analogues used in the present study.

	Analogues	Yield^a^	HPLC^b^	TLC, *R*_f_^c^	
		(%)	t*_R_* (min)	A	B
1	[D-Phe^12^, Aib^14^]CRH	41	22.79	0.22	0.39
2	[D-Phe^12^, Aib^15^]CRH	38	22.85	0.21	0.38
3	[D-Phe^12^,Cys(Et)^21^]CRH	39	23.05	0.19	0.37
4	[D-Phe^12^,Cys(Pr)^38^]CRH	37	23.28	0.20	0.36
5	[D-Phe^12^,Cys(Et)^21^, Cys(Pr)^38^]CRH	33	24.07	0.18	0.35
6	[D-Phe^12^,Cys(Pr)^21^, Cys(Et)^38^]CRH	35	23.99	0.17	0.33
7	[D-Phe^12^,Cys(Pr)^21^, Cys(Pr)^38^]CRH	36	24.32	0.17	0.34

^a^Yields were calculated on the basis of the amino acid content of the resin. All peptides were at least 98% pure.

^b^For elution conditions, see the Experimental Section.

^c^Solvent systems and conditions are reported in the Experimental Section.

**Table 3:** Binding affinities of h/r-CRH analogues.

	Analogues	Binding affinity^a^IC_50_ (nM)
	h/r-CRH	17.3
1	[D-Phe^12^, Aib^14^]CRH	226
2	[D-Phe^12^, Aib^15^]CRH	8.4
3	[D-Phe^12^, Cys(Et)^21^]CRH	28.5
4	[D-Phe^12^, Cys(Pr)^38^]CRH	35
5	[D-Phe^12^, Cys(Et)^21^, Cys(Pr)^38^]CRH	7.0
6	[D-Phe^12^, Cys(Pr)^21^, Cys(Et)^38^]CRH	3.4
7	[D-Phe^12^, Cys(Pr)^21^, Cys(Pr)^38^]CRH	17.3

^a^The values given are averages from 3 experiments performed in duplicates.

**Table 4:** Biological activity of h/r-CRH analogues.

	Analogues	Biological activity
	h/r-CRH	100% activity (Full agonist)
1	[D-Phe^12^, Aib^14^]CRH	(Partial Agonist, 60% Response max/CRH)
2	[D-Phe^12^, Aib^15^]CRH	(Partial Agonist, 60% Response max/CRH)
3	[D-Phe^12^, Cys(Et)^21^]CRH	(Full Agonist, 110% Response max/CRH)
4	[D-Phe^12^, Cys(Pr)^38^]CRH	(Partial Agonist, 60% Response max/CRH)
5	[D-Phe^12^,Cys(Et)^21^, Cys(Pr)^38^]CRH	(Partial Agonist, 70% Response max/CRH)
6	[D-Phe^12^, Cys(Pr)^21^, Cys(Et)^38^]CRH	(Partial Agonist, 60% Response max/CRH)
7	[D-Phe^12^, Cys(Pr)^21^, Cys(Pr)^38^]CRH	(Partial Agonist, 80% Response max/CRH)
